# Angular shaping of fluorescence from synthetic opal-based photonic crystal

**DOI:** 10.1186/s11671-015-0781-y

**Published:** 2015-02-28

**Authors:** Vitalii Boiko, Galyna Dovbeshko, Leonid Dolgov, Valter Kiisk, Ilmo Sildos, Ardi Loot, Vladimir Gorelik

**Affiliations:** Department of Physics of Biological System, Institute of Physics, NAS of Ukraine, Prospect Nauki 46, Kyiv, 03680 Ukraine; Laboratory of Laser Spectroscopy, Institute of Physics, University of Tartu, Ravila 14c, Tartu, 50411 Estonia; Raman Scattering Laboratory, P.N. Lebedev Physical Institute of the Russian Acad. Sci., Leninsky Prospect 53, Moscow, 119991 Russia

**Keywords:** Angular dependence, Fluorescence, Photobleaching, Photonic crystal, Refractive index, Stop band, Synthetic opal

## Abstract

Spectral, angular, and temporal distributions of fluorescence as well as specular reflection were investigated for silica-based artificial opals. Periodic arrangement of nanosized silica globules in the opal causes a specific dip in the defect-related fluorescence spectra and a peak in the reflectance spectrum. The spectral position of the dip coincides with the photonic stop band. The latter is dependent on the size of silica globules and the angle of observation. The spectral shape and intensity of defect-related fluorescence can be controlled by variation of detection angle. Fluorescence intensity increases up to two times at the edges of the spectral dip. Partial photobleaching of fluorescence was observed. Photonic origin of the observed effects is discussed.

## Background

Modification and enhancement of the fluorescence in photonic structures is important for development of optical sensors [1] and improvement of the fluorescence efficiency [2] and light harvesting ability in solar cells [3]. Most papers (see, for example, reviews [1,4]) deal with experimental and theoretical aspects of the fluorophores embedded in the multilayered films forming one-dimensional (1D) photonic crystals. The behavior of such structures is determined by the interference of light that leads to decreased reflectance and enhanced fluorescence at certain directions of observation. Fluorescence has increased intensity and higher degree of polarization at these angles, in comparison to the background emission. The increase of light intensity at certain directions may be explained by resonant coupling of fluorescence with the waveguiding leaky modes in the 1D structure that can result in shorter fluorescent lifetimes and higher radiative rates [5,6]. In particular, resonantly enhanced directional fluorescence with decreased lifetime was detected experimentally for a dye doped in 1D photonic crystals [5] and explained in terms of increased density of states near the photonic bandgap. Directional emission of light has also been reported for the multilayered films doped with quantum dots [7] and rare-earth ions [8].

Considerably less attention has been paid to the modification of light emission properties of fluorophores incorporated within three-dimensional (3D) photonic crystals, such as artificial opals composed of closely packed dielectric globules. Photonic stop zones in 3D structures have been already proved useful for blocking undesirable light emission [9]. In particular, this effect was used to avoid leakage of light from the dye-sensitized solar cells [3], to suppress the radiative channels [10], and in such a way to improve the Förster resonance energy transfer between the dye molecules situated inside the photonic structure [11].

In this work, we investigate the influence of photonic stop zones on the intrinsic fluorescence of 3D photonic crystal made of closely packed silica globules. Special attention is paid to the changes in the spectral shape of fluorescence as a function of the detection angle and the angular shift of photonic stop zone. It is demonstrated that the self-fluorescence of silica material can be enhanced at certain detection angles, near the spectral edges of the photonic stop zone.

## Methods

Preparation of photonic crystals based on synthetic opals was carried out in several steps. First, the silica globules were prepared by the hydrolysis of alkyl orthosilicate. Second, sedimentation and close-packing of these globules from the solution was achieved by centrifugation. Finally, the precipitated samples were annealed to obtain solid samples with size of several cm. Two samples (marked as 1 and 2) of compacted silica globules of slightly different sizes were selected for the study.

The samples were investigated by using optical and spectroscopic methods. SEM images of opals were obtained with EPMA SEI JXA-8200 microscope (JEOL Ltd., Akishima-shi, Tokyo, Japan). Reflectance spectra at the normal incidence were measured on JASCO V-570 spectrophotometer (JASCO International Co. Ltd., Tokyo, Japan), whereas the angular dependence of reflectance was acquired on a custom goniometric setup [12]. Fluorescence was excited with Nd:YAG laser emitting at 266 nm. Fluorescence spectra were detected by means of Andor SR303i spectrograph equipped with a CCD camera.

## Results and discussion

**Figure 1:**
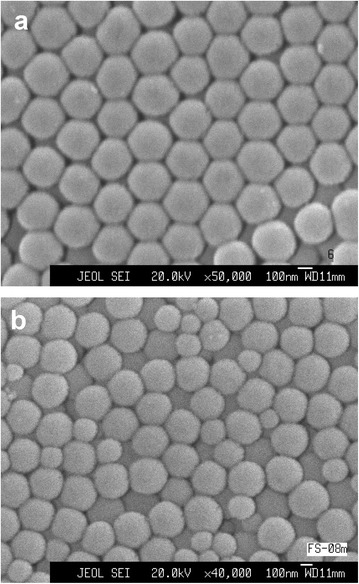
**SEM image of silica globules.** They are ordered in sample 1 **(a)** and disordered in photonic glass **(b).**

The electron micrographs of the samples (Figure 1) indicate rather uniform close-packing of nanosized silica globules (on the scale of several hundred micrometers at least). The average sizes of silica globules (determined from scanning electron microscopy (SEM) images) were 276 and 230 nm for the samples 1 and 2, respectively. According to the Raman and IR spectra [13], the globules consist of amorphous silica.

**Figure 2:**
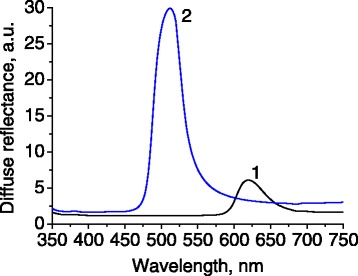
**Spectra of light reflectance for photonic samples having different sizes of silica globules.** Numbering of spectra corresponds to the numbering of samples.

Periodic spatial arrangement of silica globules results in distinct photonic properties that can be revealed in spectrally selective reflectivity. Indeed, the reflectance spectra of samples 1 and 2 (Figure 2) contain a single band of relatively strong reflectance with the peaks at 620 and 512 nm, correspondingly. The light cannot penetrate into the sample at these wavelengths, due to interference phenomena in such photonic structure.

**Figure 3:**
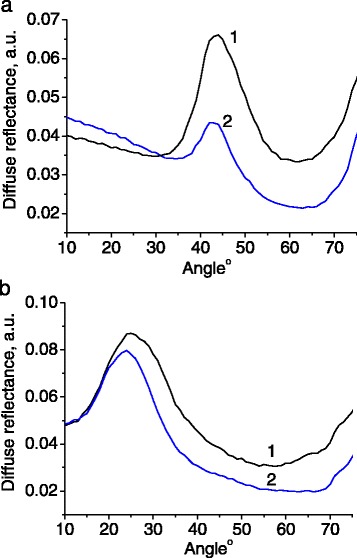
**Angular dependence of light reflectance for sample 1.** Wavelengths of incident light were 532 nm **(a)** and 593 nm **(b)**. Both s-polarized (1) and p-polarized (2) light were tested.

**Figure 4:**
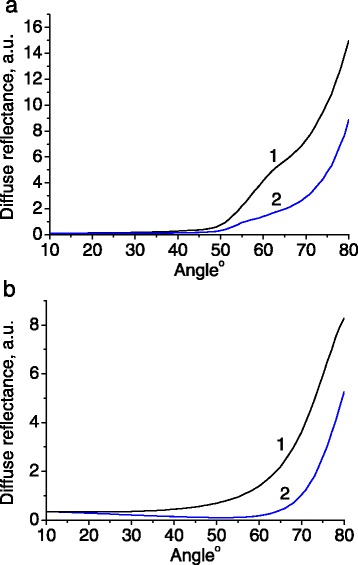
**Angular dependence of light reflectance for sample 2.** Wavelength of incident light were 402 nm **(a)** and 593 nm **(b)**. Both s-polarized (1) and p-polarized (2) light were tested.

The resonance condition depends on the direction of light propagation, and therefore, on the incidence/detection angle. The reflected light intensity was measured as a function of the detection angle for different wavelengths (Figures 3 and 4).

**Figure 5:**
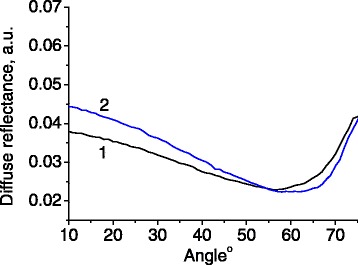
**Angular dependence of light reflectance for sample 1.** Wavelength of incident light was 402 nm. Both s-polarized (1) and p-polarized (2) light were tested.

The incident light at 532 and 593 nm lies in the spectral range of photonic stop band for sample 1. Therefore, it is reflected stronger in the angular ranges from 35° to 55° (Figure 3a) and 15° to 35° (Figure 3b), respectively. In order to prove that the angular resonances depicted in Figure 3 will disappear outside the photonic stop band, we measured the angular dependence of light reflectance at 402 nm which is clearly outside the stop band range of the sample 1 (Figure 5). On the other hand, the wavelength 402 nm enters the stop band at the range of detection angles 50° to 70° in the case of sample 2 (Figure 4a).

In principle, one could employ the Brewster law for the estimation of effective refractive index of the samples, based on the angular dependences of reflectance, measured spectrally far from the photonic stop band (Figures 4b and 5). It was revealed, however, that the diffusely reflected light restricts this possibility.

Alternatively, the effective refractive index can be calculated using a modified Bragg law, which describes the relation between the local maximum in the reflectance spectrum *λ*_max_, the corresponding angle *θ* of light detection, the size of globules *D*, and effective refractive index *n*_eff_ [14,15]:

1 $$ {\lambda}_{\max }=2{\mathrm{a}}_{\left[111\right]}\sqrt{{\mathrm{n}}_{\mathrm{eff}}^2\left(\lambda \right)\hbox{-} \mathrm{s}\mathrm{i}{\mathrm{n}}^2\theta } $$

where ${\mathrm{a}}_{\left[111\right]}=\sqrt{\frac{2}{3}}\cdot \mathrm{D}$ is the distance between the planes of closely packed silica globules in the direction [111].

For the sample 1, we have *λ*_1_ = 532 nm, *θ*_1_ = 46° (Figure 3a) and *λ*_2_ = 593 nm, *θ*_2_ = 25° (Figure 3b). Substituting these values into the Equation 1 yields a system of two equations with two unknown parameters *D* and *n*_eff_. The solution of this system gives *D* = 276 nm and *n*_eff_ = 1.38.

**Figure 6:**
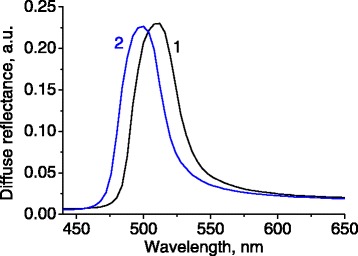
**Light reflectance spectra for sample 2 measured at the angles 10° and 20°.**

For the sample 2, we have *λ*_1_ = 512 nm, *θ*_1_ = 10° and *λ*_2_ = 500 nm, *θ*_2_ = 20° (Figure 6) yielding *D* = 230 nm and *n*_eff_ = 1.38.

Obtained refractive indices correlate well with the literature data. Accurate description of optical properties of the mixed materials, particularly porous media, is not a trivial task. A list of possible approaches, by Maxwell-Garnett, Bruggeman and Lorentz-Lorenz models, Drude, or Silberstein formula, can be found in books, for example [16], and reviews [17,18]. In our knowledge, none of the approaches mentioned above is general enough to account for the shape, size, and interconnection of pores in the real sample. Also, one can use for estimation of effective refractive index an equation known as “refractive mixing model” that is mathematically described by Birchak formula (Ref. [16], page 166). This model assumes that the refractive index of a composite mixture is an average of the indices of components weighted by their corresponding volumes:

2 $$ {n}_{\mathrm{eff}}={n}_{\mathrm{globule}}\left(\lambda \right)\cdot f+{n}_{\inf}\cdot \left(1-f\right) $$

where *n*_globule_ is a refractive index of silica, *n*_inf_ is a refractive index of the substance contained in the pores of photonic crystal (*n*_inf_ = 1 for the air), and *f* is the volume fraction of globules in the sample (*f* = 0.74 for dense hexagonal packing). Equation 2 was successfully applied earlier for the description of photonic crystals [14,15], porous films [19], and soils [20]. Effective refractive index obtained for *n*_eff_ using Equation 2 was 1.39 [14] and 1.33 [15]. These values were obtained by assuming that *n*_globule_ is equal to the refractive index of fused silica. In our opinion, it is a rough estimate, because silica nanoglobules are usually porous and, as a consequence, their refractive index could be slightly smaller than the refractive index of the fused silica.

**Figure 7:**
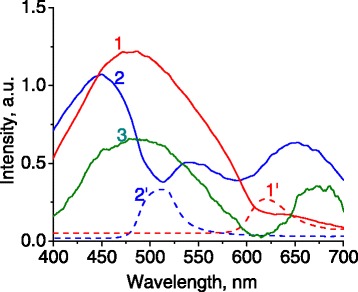
**Fluorescence spectra of photonic crystal samples with different spectral positions of stop zones.** Numbering of spectra 1 and 2 corresponds to the numbering of samples. Spectrum 3 is fluorescence of a soda lime glass slab. Spectra 1′ and 2′ are reflectance spectra of samples 1 and 2, respectively (plotted not to scale with fluorescence).

Fluorescence spectra of samples excited with UV light (*λ*_exc_ = 266 nm) consist of several broad, overlapping bands in the range of 400 to 700 nm (Figure 7).

The spectra may belong to electronic transitions in the defect centers of -Si-O-Si-O- lattice which have the energies in the visible range [21]. Particularly, the maximum at 450 nm in spectrum 2 can be associated with twofold Si-oxygen deficiency center O_2_ = Si**:** [22,23]. Fluorescence bands with maxima near 500 nm in spectra 1 and 3 can be related to the hydrogenated ≡ Si-H defects, which are formed by attaching H and OH groups to the disrupted ≡ Si• and ≡ Si-O• bonds [24]. Red luminescence in the range of 600 to 700 nm (spectra 2 and 3) can be associated with non-bridging oxygen hole centers [25] or OH groups on the surface of the silica [14].

**Figure 8:**
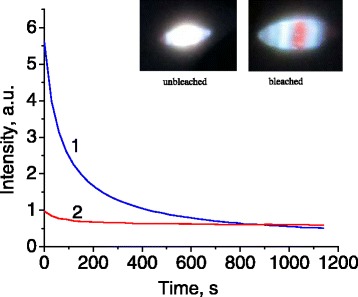
**Photobleaching of photonic crystal fluorescence in time.** Unequal kinetics of blue and red fluorescence is illustrated on the example of 450 nm (1) and 650 nm (2) spectral bands excited by steady laser irradiation at 266 nm. Images of the fluorescent spot on the surface of sample 2 in the initial and bleached states are shown in the inset.

The fluorescence emission from photonic crystals is subject to partial photobleaching. The brightness of fluorescence at the laser spot incident on the sample surface decreases essentially during the first minutes of irradiation and then changes more gradually (Figure 8). The blue fluorescence band with the maximum at 450 nm is bleaching faster than the red band with the maximum at 650 nm. As a consequence, fluorescence acquires a reddish tint at a longer exposure of the sample to UV light (Figure 8, inset).

Similar photobleaching of blue fluorescence has been reported earlier for the laser-treated silica waveguides [26]. This process may be caused by photoconversion of the silica twofold deficient centers into the E′-centers initiated by the two-photon absorption [27]. Further measurements were conducted after a preliminary UV irradiation of samples in the saturation region between 800 and 1,200 s (Figure 8), where the changes of fluorescence intensity caused by photobleaching are already minimal.

Fluorescence photobleaching may be affected by light confinement inside the photonic crystal and slow non-radiative migration of excitation between the defects in SiO_2_ material. As a consequence, the excitation energy may decrease before reaching the defect-related fluorescence center. The longer the migration time, the smaller the energy reaching the luminescent center. Therefore, less energetic red fluorescence could become predominant after a prolonged irradiation. Similar effect and its origin have been discussed before [28].

An interesting feature in the recorded fluorescence is a dip in the spectrum 2 (Figure 7), with a minimum at 507 nm that is absent in spectra 1 and 3. This spectral feature overlaps with the maximum of reflectance, caused by photonic stop zone (Figure 7, spectrum 2′). Since the photonic stop zone of the sample 1 is almost outside the spectral range of fluorescence (Figure 7, spectrum 1′), the dip in fluorescence spectrum 1 is absent. The presence of the photonic dip in spectrum 2 demonstrates fundamental opportunity to control the spectral shape of fluorescence spectrum by 3D photonic structure of opal.

**Figure 9:**
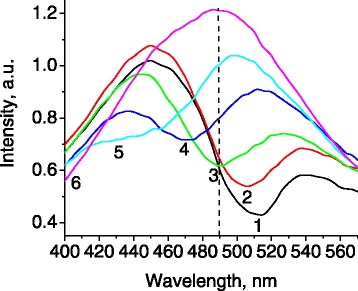
**Fluorescence spectra of sample 2 measured at different detection angles.** (1) 0°; (2) 10°; (3) 20°; (4) 30°; (5) 40°; (6) 70° (*λ*_exc_ = 266 nm).

Recording fluorescence at different angles reveals a systematic shift of the abovementioned spectral dip. It shifts toward shorter wavelengths with the increase of detection angle (Figure 9). This allows to control the fluorescence intensity in the spectral range covered by the photonic stop zone by changing detection angle. The spectral position of photonic dip in the fluorescence can be described well with Equation 1.

In addition to the angular shift of the dip in fluorescence spectra, which is clearly of photonic origin, an enhancement of the fluorescence intensity was observed near the edges of spectral dip. For example, the fluorescence intensity at the detection angle of 70° is almost two times higher than that at the angle of 0° at the wavelength of 490 nm (marked by dashed line in Figure 9). We suppose that such angular enhancement in fluorescence also has photonic nature and is caused by blue shift of the photonic stop band at large angles. Similar effect was described for the light transmitted through a thin photonic crystal film [29]. The overlap of diffractional resonances associated with different systems of crystallographic planes would also lead to a redistribution of light intensity on the edges of the stop bands, which is visible for the transmitted light in Figures 2 and 3 in Ref. [29].

**Figure 10:**
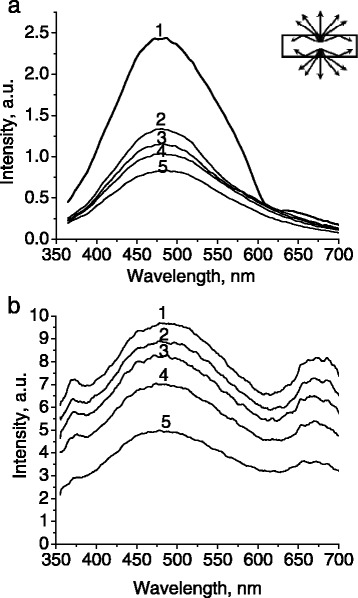
**Fluorescence spectra of photonic glass (a) and soda lime glass slab (b), measured at different angles.** (1) 0°; (2) 40°; (3) 50°; (4) 60°; (5) 70°. The inset depicts total internal reflection of light at high angles.

It should be noted that fluorescence intensity decreases at increasing of the observation angle for the fluorescent samples without a photonic superlattice, such as photonic glasses with disordered silica globules (Figure 10a) and reference slabs of soda lime glass (Figure 10b). Fluorescence emission experiences stronger refraction at high observation angles and higher reflectance at the air-glass interfaces, thus being unable to leave the sample (Figure 10a, inset).

## Conclusions

We demonstrated that intrinsic fluorescence of opal-based photonic crystals can be influenced by photonic stop zone. A decrease of the fluorescence at the wavelengths within the photonic stop band and its increase near the edges of stop band were observed. This effect could be proved by comparison of the fluorescent spectra detected at different angles, despite the undesirable photobleaching of the samples.
